# Engineering Decavalent, Sperm‐Binding Laminin‐IgG Hybrid Antibodies for Potent Non‐Hormonal Contraception

**DOI:** 10.1002/advs.202506272

**Published:** 2025-07-23

**Authors:** Alison Schaefer, Keiichiro Kushiro, Bhawana Shrestha, Yong Zhu, Alysha Panjwani, Lauren Dawson, Haley Flowers, Kathleen L. Vincent, Samuel K. Lai

**Affiliations:** ^1^ Department of Biomedical Engineering University of North Carolina – Chapel Hill Morrisville NC USA; ^2^ Mucommune LLC Morrisville NC USA; ^3^ Department of Immunology and Microbiology University of North Carolina – Chapel Hill Morrisville NC USA; ^4^ University of Texas Medical Branch at Galveston Galveston TX USA; ^5^ Division of Pharmacoengineering and Molecular Pharmaceutics Eshelman School of Pharmacy University of North Carolina – Chapel Hill Morrisville NC USA

**Keywords:** antibodies, cervicovaginal antibody delivery, non‐hormonal contraception

## Abstract

Nearly half of pregnancies globally are unintended, reflecting the current unmet need in safe, effective non‐hormonal contraception. While anti‐sperm IgM is responsible for infertility in many women, stability and bioprocessing challenges with IgM make them ill‐suited for non‐hormonal contraception. Similarly, IgG lacks sufficient sperm agglutination potencies. To overcome these shortcomings, a novel, multivalent mAb platform is developed, based on fusing the Laminin 511 heterotrimerization domains to Fabs and IgG1‐Fc, allowing for tuning of Fab valency (from 2–12) with molecular specificity. ‘LamH10’, the most potent mAb among the panel of polyvalent antibodies against CD52g on human sperm, comprises 10 Fabs per molecule, and achieves nanomolar potencies at physiological temperatures and retains stability in the acidic environment of human cervicovaginal mucus. In sheep, LamH10 reduced progressively motile sperm in the vagina by >99% within 2 min at just 33 µg per sheep. LamH10 utilizes the same conventional bioprocessing as IgGs, and can be formulated into rapidly dissolving tablets for on‐demand contraception, achieving 100% sperm agglutination within minutes of vaginal dosing. The laminin‐IgG hybrid platform not only forms the basis of the most potent biologic for nonhormonal contraception to date, but also represents a promising platform for multivalent mAbs for other applications.

## Graphical abstract

This study investigates a highly multivalent mAb platform fusing Laminin‐511 heterotrimerization domains with Fabs and IgG1‐Fc as a non‐hormonal contraceptive. The most potent molecule ‘LamH10’, possessing 10 Fabs, rapidly achieved >99% reduction in progressively motile sperm at just 33µg per dose in sheep, making the laminin‐IgG hybrid platform the basis of the most potent biologic for nonhormonal contraception to date.

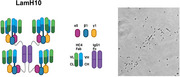

## 1 Introduction

Unintended pregnancy is associated with poorer maternal and child health outcomes,^[^ ^1^ ^]^ and represented a public health burden of nearly $21 billion in the US alone as of 2010.^[^ ^2^ ^]^ Despite the widespread availability of contraceptive methods, nearly half of all pregnancies in the U.S. remain unintended or unwanted.^[^ ^3^, ^4^ ^]^ A key factor in limited contraceptive use is the hormonal nature of the vast majority of contraceptive options available; although generally safe and effective, many women simply prefer non‐hormonal options due to real or perceived side‐effects.^[^ ^5^, ^6^, ^7^ ^]^ Substantial percentages also have medical contraindications.^[^ ^8^, ^9^ ^]^ The only long‐acting, reversible non‐hormonal contraceptive available is the copper IUD, which is often discontinued due to side effects such as dysmenorrhea and heavy menstrual bleeding.^[^ ^10^, ^11^, ^12^ ^]^ The currently available on‐demand, female‐initiated non‐hormonal contraceptives either increase the risk of STI transmission,^[^ ^13^, ^14^, ^15^ ^]^ are messy to administer (gels, cream), and/or are non‐discrete. Thus, we believe there is a major unmet need for alternative non‐hormonal contraceptives.

Among the different potential therapeutic classes, we believe anti‐sperm antibodies (ASAs), the basis of immune‐infertility in many women,^[^ ^16^ ^]^ offer the most promising means of safe and effective non‐hormonal contraception. ASA can preclude otherwise motile sperm from reaching the egg, either by clumping sperm into clusters too large to swim through mucus^[^ ^17^, ^18^ ^]^ (i.e., agglutination), or by crosslinking sperm to mucins via multiple low‐affinity Fc‐mucin bonds (i.e., muco‐trapping).^[^ ^19^, ^20^, ^21^, ^22^ ^]^ One such ASA (H6‐3C4,^[^ ^23^ ^]^ or ‘HC4’) has been extensively investigated for this purpose. Originally an IgM ASA isolated from an immune infertile woman,^[^ ^24^ ^]^ HC4 binds a unique glycoform of CD52 (CD52g)^[^ ^25^ ^]^ found ubiquitously only on cells in the male reproductive tract and is generally absent in other tissues in women.^[^ ^25^, ^26^, ^27^ ^]^ To facilitate bioprocessing for clinical development, HC4 has been reformatted as IgG monoclonal antibody (mAb),^[^ ^28^ ^]^ and was recently tested as a vaginal film in an exploratory clinical study.^[^ ^29^ ^]^ Since IgG is generally a poor agglutinating molecule compared to polymeric species such as IgM,^[^ ^24^ ^]^ there is considerable interest in advancing higher valency mAb formats that possess much greater potencies than IgG while retaining the stability and manufacturing ease of IgG.^[^ ^19^, ^20^, ^21^ ^]^

In our pursuit of engineering more potent ASA for non‐hormonal contraception, we discovered here that HC4‐IgG quickly unbinds from CD52g on sperm at physiological temperature (i.e., 37°C). As the original IgM is known to cause immune infertility in vivo, we hypothesized that increased Fab valency could generate sufficient binding avidity to overcome the temperature‐induced Fab unbinding from sperm. While exploring different multivalent mAb platforms, we uncovered key shortcomings that make them either ill‐suited for vaginal applications. This led us to develop a novel multivalent mAb platform, based on linking laminin‐511 trimerization domains^[^ ^30^ ^]^ to IgG1‐Fc, and co‐express with other Fab‐trimerization domain conjugates. We report here the development of this laminin‐IgG hybrid platform, as well as in vitro and in vivo studies supporting the lead candidate, LamH10, as an exceptionally promising mAb for non‐hormonal contraception.

## 2 Results

### 2.1 Sufficient Multivalency is Required to Maintain Sperm Agglutination Potency at Physiological Temperature

**Figure 1:**
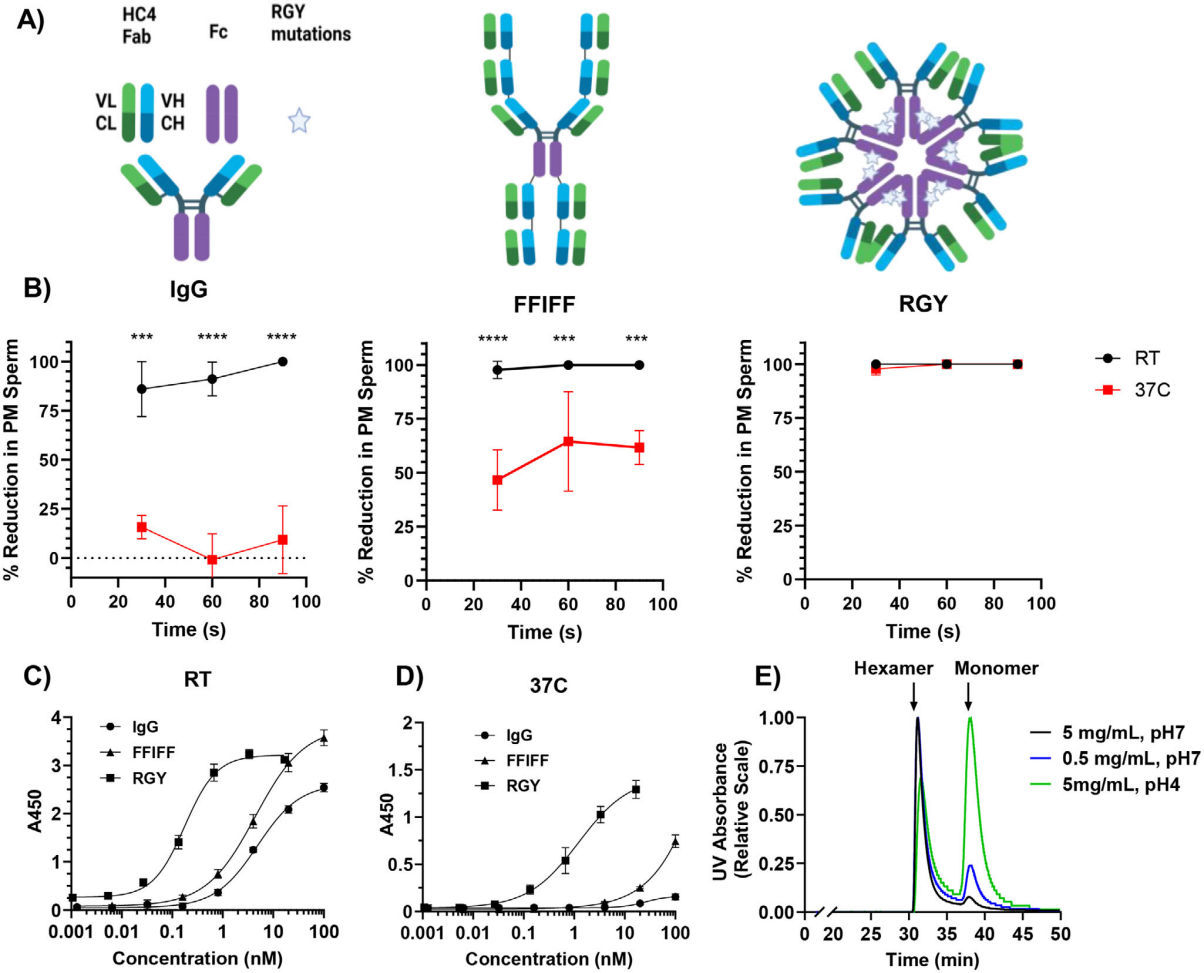
Comparison of sperm‐agglutinating potency of IgG, FFIFF, and RGY formats. (A) Schematic illustration of IgG, FFIFF, and RGY mAb formats (left to right). (B) Sperm agglutination with Ab at 10µg mL^−1^ as assessed by agglutination kinetics assay against purified sperm at a final concentration of 20×10^6^ sperm mL^−1^, performed at RT and at 37°C (n = 3 semen samples from unique donors). (C, D) Binding affinity assessed by whole‐sperm ELISA at (C) RT and (D) 37°C. Samples were plated in triplicate and averaged. (E) Size exclusion chromatography profile for RGY mAb illustrating the relative abundance of hexamer and monomer populations as a function of concentration and pH. P‐values were calculated by two‐way ANOVA with post‐hoc Tukey's test on arcsin‐transformed data. ^****^ *p* < 0.0001, ^***^ *p* < 0.001. Lines and error bars indicate arithmetic mean values and standard deviation.

The fifth edition of the WHO laboratory manual for semen analysis,^[^ ^31^ ^]^ in use until 2021, allowed for fertility assessments of sperm motility at either room temperature or 37°C. As a result, prior studies assessing sperm‐acting contraceptives have frequently done so at room temperature.^[^ ^32^, ^33^, ^34^, ^35^ ^]^ Upon using a carefully thermally‐calibrated microscopy setup, it became apparent that the HC4‐IgG (**Figure** **1**A) had greatly reduced sperm‐agglutinating activity at physiological temperature. While HC4‐IgG effectively agglutinated sperm at 10 µg mL^−1^ at room temperature, consistent with prior studies^[^ ^19^, ^20^, ^21^ ^]^ (Figure 1B), HC4‐IgG lost essentially all agglutination activity at the same concentration at 37°C (Figure 1B). The loss of sperm binding was reproduced via temperature‐controlled ELISA assays, with HC4‐IgG exhibiting an IC_50_ of ≈5nM at RT (Figure 1C) and binding below the limit of detection at 37°C (Figure 1D).

As the Fab variable region was derived from an IgM sequence isolated from an immune‐infertile woman,^[^ ^24^ ^]^ we hypothesized that the loss in sperm binding and agglutination observed for HC4‐IgG should be resolved with a suitable multivalent HC4 mAb. Previously, we had developed a decavalent molecule comprised of ten total Fabs and one Fc, where additional Fabs are fused serially to the N‐ and C‐terminus of the heavy chains of HC4‐IgG. The molecule, which we termed FFIFF (Figure 1A) ^[^ ^20^ ^]^ in reflection of its molecular arrangement, possessed at least 16‐fold greater sperm agglutination potency than the parent HC4‐IgG. Unfortunately, while capable of mediating more stable binding and sperm agglutination than HC4‐IgG, its overall potency was nearly halved when the temperature was raised to 37°C. The reduced binding is also confirmed via temperature‐controlled ELISA assays. At RT, the EC_50_ of FFIFF was 4.4 nM (Figure 1C), which increased to beyond the measurement range at 37°C (Figure 1D).

We note that, due to the Fab arrangements, most Fab domains on FFIFF are likely to experience substantial steric hindrance near the N‐terminus of the heavy chain, potentially contributing to reduced binding valency and occupancy of available Fabs. This contrasts sharply with conventional IgM, where the 10–12 Fab domains in each IgM are free from any such steric obstruction. This led us to first explore other multivalent IgG with structural formats similar to IgM. We specifically focused on IgGs with Fc mutations known as RGY (Figure 1A) ^[^ ^36^ ^]^ that allow self‐hexamerization via non‐covalent associations between different IgG‐Fc. As expected, the resulting HC4‐IgG‐RGY hexamer (i.e. HC4‐RGY) exhibited superior sperm‐agglutination (Figure 1B) and sperm binding (Figure 1C) at RT comparable to FFIFF. In good agreement with our hypothesis, HC4‐RGY also exhibited comparable sperm agglutination potency at 37°C as RT, and a markedly less reduction in EC_50_, which was ≈0.17nM at RT (Figure 1C) and ≈1.22 nM at 37°C (Figure 1D). These results underscore that suitably engineered multivalent IgGs can overcome the increased HC4‐Fab unbinding at elevated temperatures.

Despite the excellent potency at 37°C, there are critical shortcomings with the RGY‐platform that limits its use for vaginal applications. Specifically, RGY‐mediated assembly is non‐covalent, and thus the ratio of RGY hexamers and monomers represents an equilibrium that can be altered by the environment. Indeed, at neutral pH, HC4‐RGY shifted from ≈10% monomer at 5 mg mL^−1^ to ≈25% monomer at 0.5 mg mL^−1^. Similarly, after incubation at pH 4 for 1 hr, the monomer fraction was increased to ≈62% of the mAb (Figure 1E). These results are in good agreement with prior studies on the biophysical characteristics of the RGY format,^[^ ^37^ ^]^ and make HC4‐RGY incompatible with delivery at low concentrations to the vagina, where pH typically ranges between 3–5 due to acidification by resident Lactobacilli.

### 2.2 Design and Characterization of Highly Multivalent mAbs with Unencumbered Fabs Using Laminin Trimerization Domains

The excellent sperm agglutination potency observed with HC4‐RGY and the strong biophysical stability observed with FFIFF motivated us to develop a multivalent IgG format that did not require fusing additional Fabs along the N‐terminus of the heavy chains, while also avoiding non‐covalent self‐assembly of multiple IgG molecules. These design requirements led us to explore introducing multimerization domains present in various human proteins, which could be fused between the C‐terminus of a Fab and the N‐terminus of the Fc region; additional binding domains can then be incorporated by expressing additional conjugates of Fab fused to a trimerization domain. We first screened homotrimeric domains present in collagen XV and collagen XVIII, which have been used previously to display V_HH_ and scFV without an Fc domain.^[^ ^38^ ^]^ However, this resulted in distinct populations with multiple peaks on chromatography (Figures S1A,B, Supporting Information), likely due to limited control over the assembly. We next screened three heterotrimeric domains, two of which produced poorly and assembled mixed populations after fusion into the IgG (Figures S1C,D, Supporting Information). In contrast, molecules utilizing the heterotrimerization domain from laminin‐511,^[^ ^30^ ^]^ composed of unique alpha, gamma, and beta domains, which is further stabilized by disulfide bonds when complexed,^[^ ^39^ ^]^ yielded a single peak consistent with the expected size of a properly assembled molecule.

**Figure 2:**
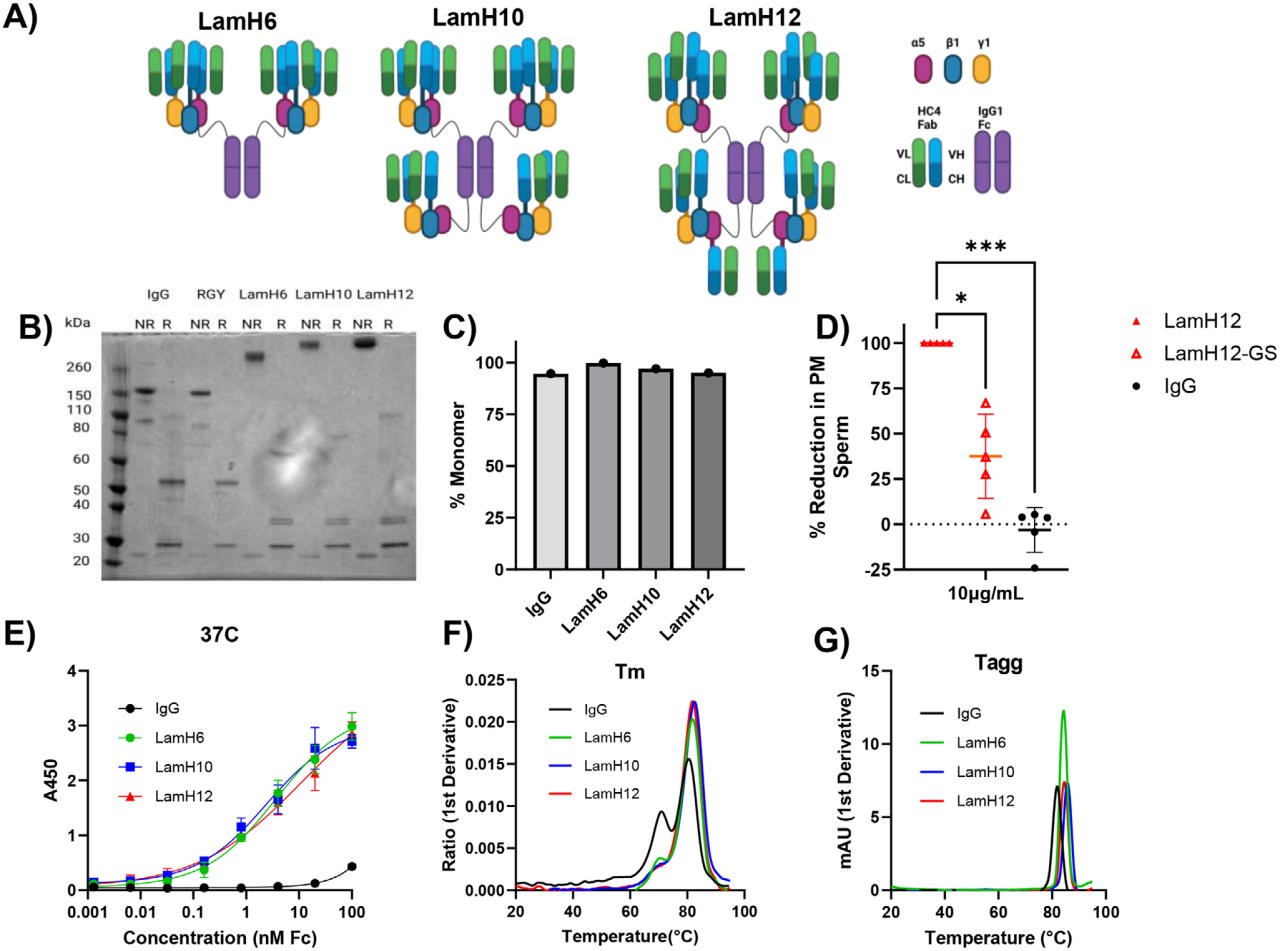
Characterization of laminin‐trimerized HC4 (i.e. LamH*n*) mAbs. (A) Schematic illustration of different laminin‐IgG multivalent hybrids possessing 6 (LamH6), 10 (LamH10), or 12 (LamH12) HC4 Fabs, respectively. (B) SDS‐PAGE analysis of various LamH*n* mAbs in either non‐reduced (NR) or reduced (R) conditions. (C) Relative monomer content for each LamH*n* mAb as measured by SEC‐MALS. HC4 IgG is included as a control. (D) Relative reduction in PM sperm fractions achieved with LamH12 utilizing a hinge linker between the HC4 Fab and laminin trimerization domains, and LamH12 utilizing the flexible (G_4_S)_6_ linker to link HC4 Fabs to laminin trimerization domains (“LamH12‐GS”). HC4‐IgG and HC4‐RGY are included as controls. PM sperm fractions were determined at 90s and 10µg mL^−1^ final mAb concentrations, and encompasses 5 independent experiments on unique semen samples. (E) Binding affinity of various mAbs assessed by whole‐sperm ELISA at 37°C. Samples were plated in triplicate and averaged. (F‐G) Stability of various LamH*n* mAbs as assessed by (F) melting temperatures (Tm) and (G) aggregation temperatures (Tagg) measured by nanoDSF. The experiment was performed in triplicate and averaged. P‐values were calculated using the Kruskal‐Wallis test followed with post‐hoc Dunns's multiple comparisons test. ^***^ *p* < 0.005, ^*^ *p* < 0.05. Lines and error bars indicate arithmetic mean values and standard deviation.

We next designed a panel of laminin‐511 IgG hybrids with different numbers of HC4‐binding domains (LamH*_n_*), including either 6 (LamH6), 10 (LamH10), or 12 (LamH12) HC4‐Fab domains (**Figure** **2**A). All three LamH*_n_* constructs demonstrated a clean monomer band and were reducible to correctly sized individual protein chains in an SDS‐PAGE (Figure 2B). The correct size of the monomer and overall homogeneity were further confirmed by SEC‐MALS, with all constructs demonstrating similar homogeneity to the parent IgG (Figure 2C; Figure S2, Supporting Information). Notably, the choice of linker was critical to effective sperm agglutination at 37°C (Figure 2D). Even with 12 Fabs, LamH12 constructs utilizing glycine‐serine linkers between the Fab and trimerization domains (LamH12‐GS) exhibited less than half of the sperm agglutination potency as essentially the same LamH12 format utilizing linkers composed of the upper‐hinge region of a native IgG_1_ (LamH12). Binding affinities, as measured by ELISA at 37°C, were found to be 3.7nM, 2.3nM, and 11.2nM for LamH6, LamH10 and LamH12, respectively (Figure 2E). Room temperature ELISA of LamH10 exhibited an EC50 of 0.07nM (Figure S3, Supporting Information). Additionally, all 3 constructs exhibited similar thermal stability properties to the parent IgG, with melting temperatures T_m1_ ≥ 71.2°C and T_m2_ ≥ 80.3°C (Figure 2F), and aggregation temperatures T_agg_ ≥ 80°C (Figure 2G).

### 2.3 LamH10 Rapidly Agglutinates Both Purified Motile Sperm and Sperm in Whole Semen

**Figure 3:**
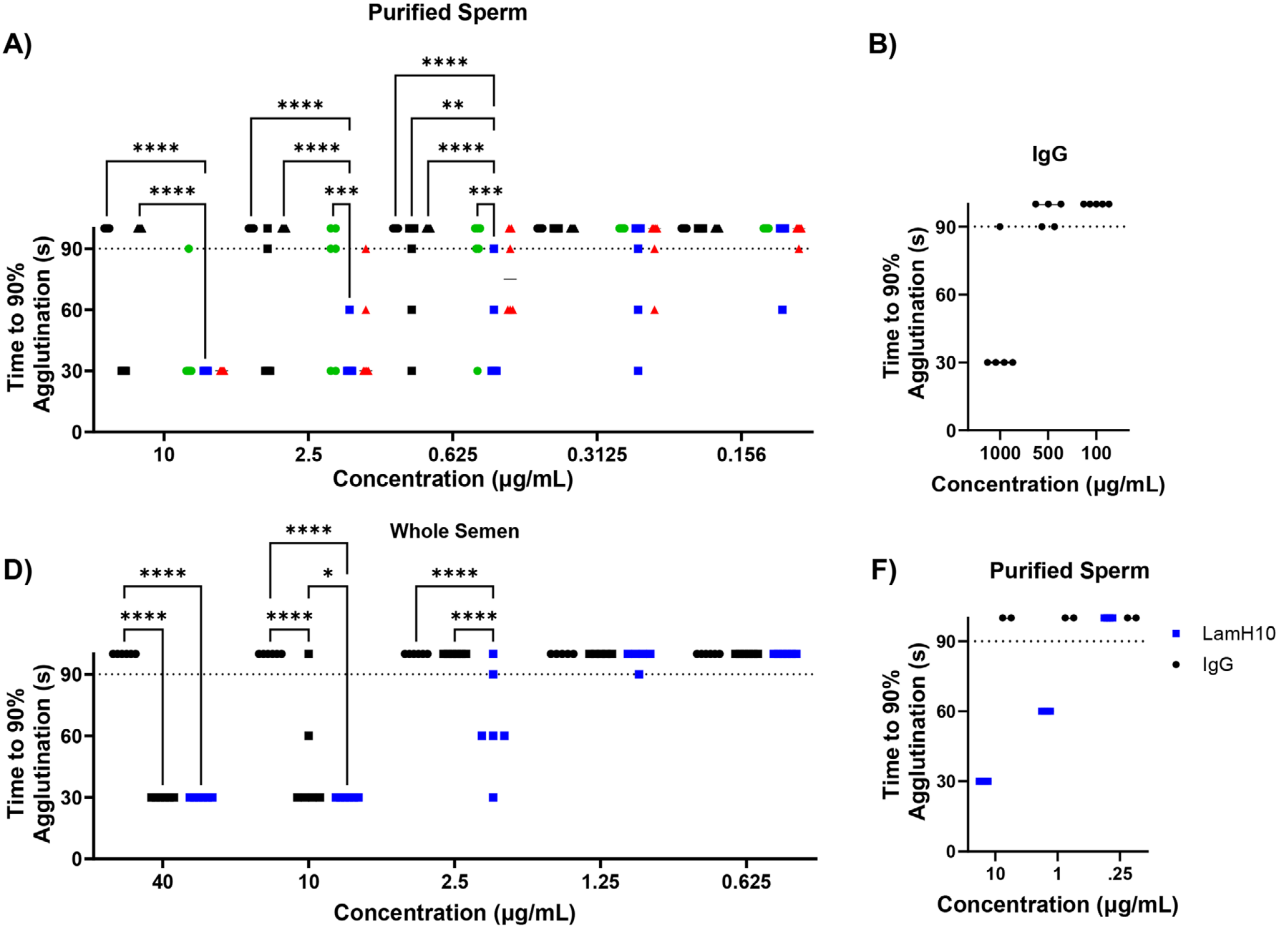
LamH*n* mAbs induce potent sperm agglutination at 37°C against purified sperm and whole semen. (A) Sperm agglutination kinetics of parent HC4‐IgG, HC4‐RGY hexamer, LamH6, LamH10, and LamH12 were quantified by measuring the time to achieve at least 90% agglutination of PM sperm, compared to sperm‐handling media control. Assay was performed using purified sperm at a final concentration of 20 ×10^6^ sperm mL^−1^ (n = 6 samples) (B) Sperm agglutination of parent IgG at 37°C at high concentration against purified sperm. (n = 5 samples)(C) Sperm agglutination kinetics of LamH10 compared to RGY and parent IgG when conducted using whole semen (n = 6 samples) (D) Independent site evaluation of agglutination potency of LamH10 compared to parent IgG against purified sperm (n = 2 semen samples). Measurements in each sample were performed in duplicate and averaged. P‐values were obtained by two‐way ANOVA with post‐hoc Tukey's multiple comparisons test. ^**^ *p* < 0.01, ^***^ *p* < 0.001, ^****^ *p* < 0.0001. Lines represent the median value in panel A; lines and error bars indicate arithmetic mean values and standard deviation in panel B. Dotted lines indicate the highest time or concentration tested, and points above represent samples that did not achieve 90% agglutination under any tested condition.

Sperm is thought capable of ascending to the endocervix as quickly as 90 s post‐coitus^[^ ^40^ ^]^; thus, we believe rapid sperm agglutination is likely an important correlate for contraceptive efficacy. We quantified the kinetics of sperm agglutination by measuring the reduction in PM sperm over time immediately following mixing of sperm and various mAbs via Computer‐Assisted Sperm Analysis (CASA), with the key metric defined as ≥90% reduction in PM sperm within 90s. We initially tested potency against purified (washed) sperm at a concentration of 20×10^6^ sperm mL^−1^. At 37°C, the parent IgG reduced PM sperm by less than 10% on average after 90s at the highest concentration tested (10 µg mL^−1^) across 6 semen samples (**Figure** **3**A; Figures S4 and S5, Supporting Information), and had no observable effect at lower concentrations. In order to agglutinate consistently at 37°C, the concentration of IgG had to be increased 100‐fold to 1 mg mL^−1^ (Figure 3B). In contrast, RGY induced nearly 100% reduction in PM sperm within 30s at 10 µg mL^−1^, achieved ≥90% reduction in PM sperm within 90s in 5 of 6 semen samples tested at 2.5 µg mL^−1^, and in 3 of 6 semen samples tested at 0.625 µg mL^−1^ (Figure 3A; Figures S4 and S5, Supporting Information).

Despite only 6 HC4 Fab domains, the sperm‐agglutination potency of LamH6 was nearly on par with HC4‐RGY, with 12 Fabs per molecule. LamH10 exhibited considerably greater sperm agglutination and was the most potent sperm agglutinator, mediating highly effective sperm agglutination in all 6 semen samples tested at 0.625 µg mL^−1^ (Figure S6, Supporting Information). Indeed, in all semen samples tested, LamH10 captured ≥90% of sperm within just 30s at 10 µg mL^−1^, within 60s at 2.5 µg mL^−1^, and within 90s at 0.625 µg mL^−1^. The difference in sperm agglutination was statistically significant over LamH6 at 2.5 µg mL^−1^, and over both HC4‐RGY and LamH6 at 0.625 µg mL^−1^ (Figure 3A; Figures S4 and S5, Supporting Information). Interestingly, LamH12 was slightly less effective than LamH10 despite the 2 additional Fabs, capturing 90% of sperm within 90s in only 4 out of 6 samples at 0.625 µg mL^−1^. Based on these results, we moved forward with LamH10 as the best candidate in future assays.

We next assessed the sperm agglutination kinetics of LamH10 against whole semen. Notably, the concentration of sperm in whole semen is variable, ranging from 20‐ 100×10^6/mL in the samples examined, and also contains substantial extracellular vesicles with CD52g ^[^ ^41^ ^]^ in the seminal plasma. Not surprisingly, the parent HC4‐IgG was unable to agglutinate meaning fractions of sperm at 37°C in whole semen, even at the highest tested concentration of 40 µg mL^−1^ (Figure 3D; Figures S5 and S7, Supporting Information). HC4‐RGY captured at least 90% of PM sperm within 30s at 40 µg mL^−1^ in all 6 samples, 90% within 90s in 5 out of 6 samples at 10 µg mL^−1^, but was almost entirely ineffective at 2.5 µg mL^−1^ (Figure 3C; Figures S5 and S7, Supporting Information). LamH10 remained more effective than HC4‐RGY in whole semen, able to capture nearly 100% of sperm within 30s at both 40 and 10 µg mL^−1^ in all samples, and at least 90% capture within 90s in 5 out of 6 samples down to 2.5 µg mL^−1^ in whole semen (Figure 3C; Figures S5 and S7, Supporting Information). The ≈fourfold shift in mAb concentrations necessary to support potent sperm agglutination in whole semen compared to purified sperm is consistent with our earlier findings with FFIFF.^[^ ^20^ ^]^ Additionally, the agglutination potency of LamH10 against purified sperm was independently confirmed by a different scientist at a different site (KK; Figure 3D).

We further assessed the stability of LamH10 after exposure to whole, undiluted CVM at native pH overnight at 37°C. The supernatant of the sample was then centrifuged to remove mucins, and the remaining mAb in the supernatant was assessed for agglutination potency against whole semen. Although there was a modest reduction in the agglutination potency, LamH10 was at least as stable as the parent IgG in CVM (Figure S8A, Supporting Information), indicating that the laminin trimerization domains are not prone to acidic or enzymatic cleavage from CVM. Additionally, LamH10 remained stable and effective in solution after being held at 37°C for at least 2 weeks (Figure S8B–D, Supporting Information). SEC analysis of LamH10 at 5 and 0.5 mg mL^−1^ and at pH 4 further showed that LamH10 does not undergo concentration and pH‐dependent dissociation as does RGY (Figure S8E, Supporting Information).

### 2.4 LamH10 More Effectively Reduces Sperm Escape from Ejaculate than IgG and RGY Hexamer

Generally, bulk mixing of semen and cervicovaginal mucus (CVM) is limited due to their viscoelastic rheological properties^[^ ^40^ ^]^; sperm must swim out of the ejaculate and toward the cervical os. As a separate independent assessment of sperm agglutination, we utilized a modified sperm escape assay to measure the quantity of sperm that escape the ejaculate when exposed to a layer of media containing ASAs.^[^ ^20^, ^21^ ^]^ The parent IgG exhibited an average of only ≈26% reduction in escaped PM sperm at 40 µg mL^−1^ compared to no Ab control, and on average, no observable reduction at lower concentrations across 6 semen samples tested (Figure S9A,B, Supporting Information). Consistent with the sperm agglutination kinetics study, RGY reduced escaped PM sperm by ≈97% at 40 µg mL^−1^, by ≈77% at 10 µg mL^−1,^ and by only ≈16% at 0.625 µg mL^−1^. LamH10 again offered the most effective sperm agglutination among all mAbs tested, achieving 100% reduction in escaped PM sperm at 40 µg mL^−1^, ≈97% reduction at 10 µg mL^−1^, and still ≈50% reduction at 0.625µg mL^−1^ across the 6 semen samples tested. We further directly compared the reduction in efficacy at preventing sperm escape at 37°C and at RT. Although LamH10 does exhibit some minor loss in sperm agglutination potency at elevated temperature, it retains the sperm agglutination activity measured at RT to a greater extent than either the parent HC4‐IgG or HC4‐RGY (Figure S9C, Supporting Information).

### 2.5 LamH10 Preserves Muco‐Trapping Fc‐Effector Function

**Figure 4:**
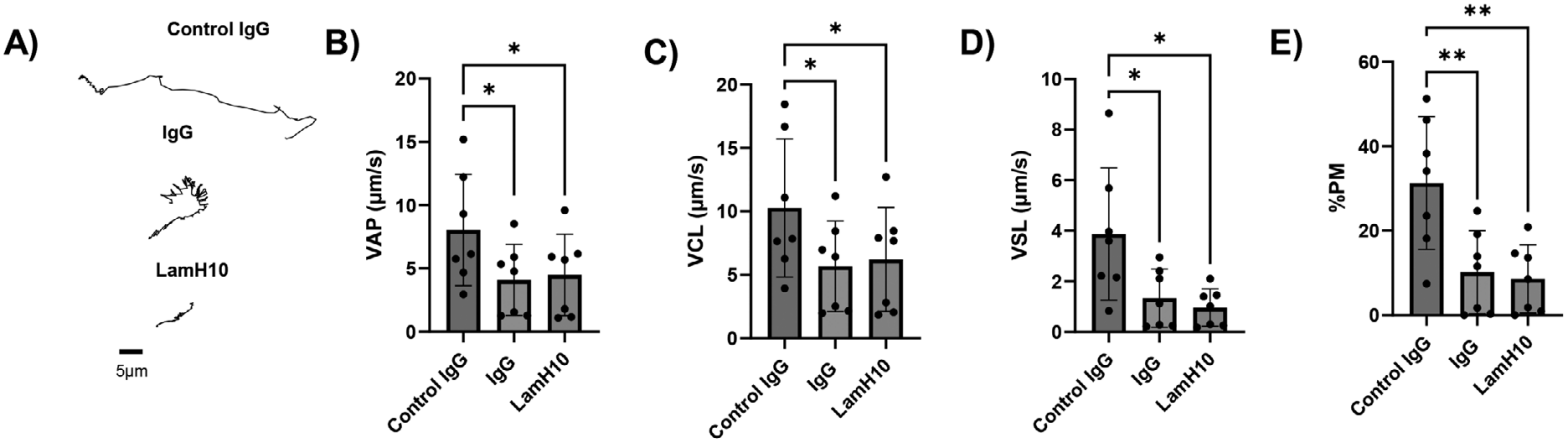
LamH10 maintains Fc muco‐trapping activity against individual motile sperm. (A) Representative traces of semen in native CVM or CVM treated with HC4‐IgG or LamH10. The mobility of the traces is within one standard deviation of the mean values in each condition. Average (B) velocity, (C) curvilinear velocity, and (D) straight‐line velocity of tracked sperm in each condition. (E) Percentage of sperm classified as PM. Data obtained from n = 6 semen samples from unique donors. P‐values were obtained by matched‐samples one‐way ANOVA with post‐hoc Holm‐Sidak test. ^*^ *p* < 0.05, ^**^ *p* < 0.01. Lines and error bars indicate arithmetic mean values and standard deviation.

Previous work has observed the “shaking phenomenon”, where ASAs can trap individual sperm in mucus despite the vigorous motion of the sperm flagellum.^[^ ^18^, ^19^, ^20^, ^21^, ^22^, ^42^ ^]^ This phenomenon is dependent on the Fc portion of Ab forming adhesive crosslinks with mucins,^[^ ^22^, ^43^ ^]^ and is consistent with prior observations from our lab in which Ab can immobilize viruses in mucus by low‐affinity crosslinks between the Fc domain and mucins.^[^ ^44^, ^45^, ^46^, ^47^, ^48^ ^]^ We thus sought to assess whether our laminin‐based IgG hybrid molecules can retain the muco‐trapping potencies against individual spermatozoa, given that the additional Fabs trimerized via the laminin‐511 domain to the C‐terminus of Fc likely poses steric hindrance to Fc access. We used high spatiotemporal video microscopy to quantify the motility of hundreds of individual spermatozoa in different conditions (**Figure** **4**A). Both the parent IgG and LamH10 reduced the velocity of the average path (Figure 4B) and curvilinear velocity (Figure 4C) ≈twofold compared to control IgG, and reduced the straight‐line velocity (Figure 4D) and the overall % PM sperm (Figure 4E) at least threefold. These results suggest LamH10 possesses comparable muco‐trapping potencies as IgG mAbs despite its considerably greater diameter.

### 2.6 LamH10 Effectively Reduces PM Sperm in the Sheep Vagina

**Figure 5:**
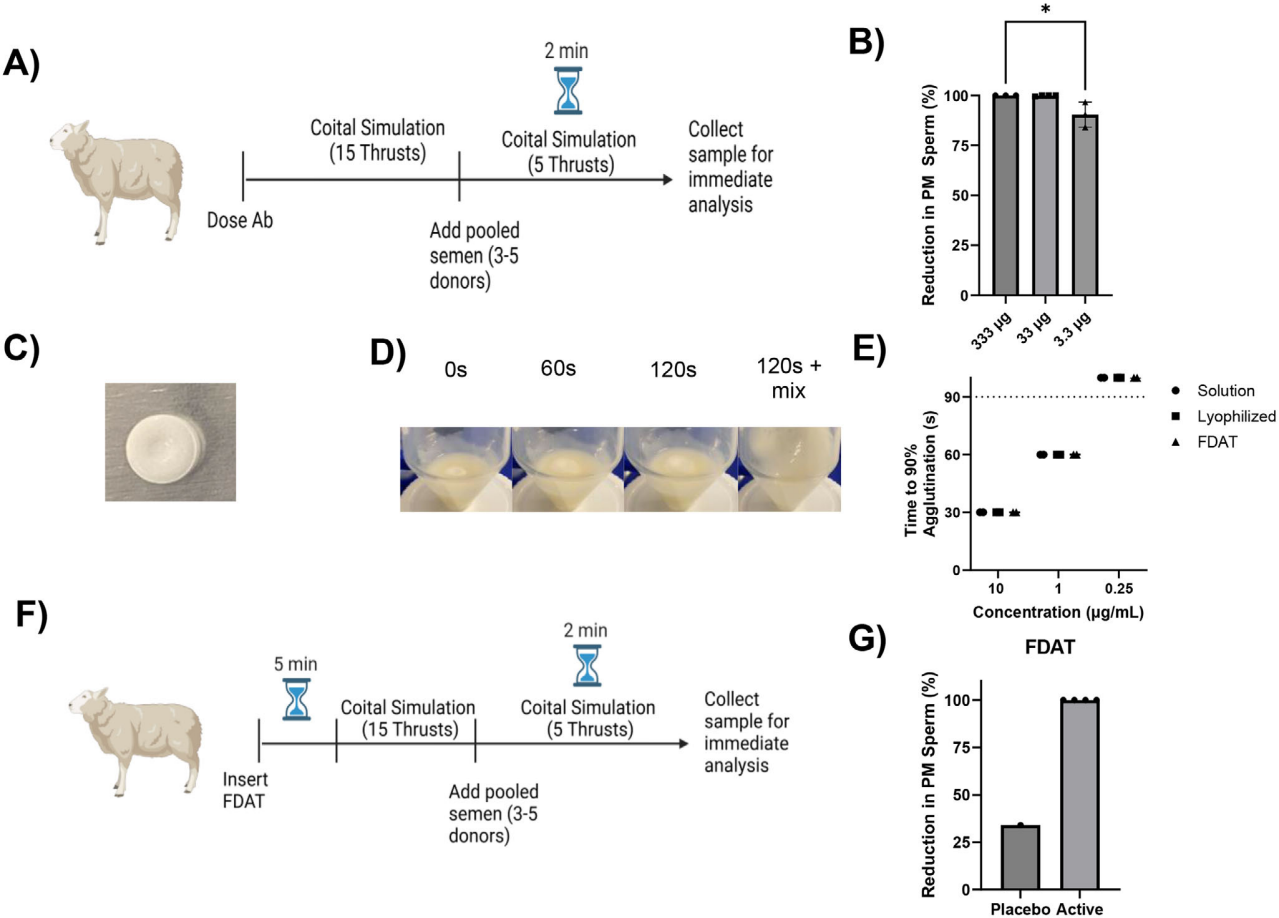
LamH10 demonstrates potent sperm agglutination in sheep. (A) Schematic of study design. (B) Reduction in PM sperm compared to PBS control for LamH10 dosed at different concentrations. (C) Photograph of LamH10 Fast‐Dissolving Ab Tablet (FDAT). (D) Dissolution kinetics of FDAT in CVM. (E) Sperm agglutination potency of LamH10 in solution compared to potency after reconstitution from lyophilized powder or FDAT (n = 2 samples). (F) Schematic of sheep study design evaluating the effectiveness of LamH10 in agglutinating sperm within 5 mins of vaginal dosing. (G) Reduction in PM sperm compared to PBS control for placebo vs. LamH10 FDAT. P‐values were obtained by Kruskal‐Wallis followed by post‐hoc Dunn's test. ^*^ *p* < 0.05, Lines and error bars indicate arithmetic mean values and standard deviation. In panel G, statistical analysis was not possible due to a lack of variance between samples.

As the unique glycoform of CD52g targeted by H6‐3C4 is found only in human and chimpanzee sperm,^[^ ^49^ ^]^ there is no suitable animal model available to directly assess contraceptive efficacy. Instead, we evaluated the in vivo effectiveness of LamH10 using a sheep model of human post‐coital test (PCT),^[^ ^50^ ^]^ which has proven effective predictors of eventual contraceptive efficacy.^[^ ^51^, ^52^, ^53^ ^]^ Sheep is typically used for assessing vaginal products due to physiological and geometrical similarity of the female reproductive tract to human.^[^ ^54^, ^55^ ^]^ Similar to earlier studies,^[^ ^19^, ^20^, ^21^ ^]^ we instilled either PBS or defined quantities of LamH10 into the sheep vagina, followed by simulated intercourse with a vaginal dilater (15 strokes), instillation of whole human semen, and further brief simulated intercourse (5 strokes) (**Figure** **5**A). Finally, the sample was recovered from the sheep vagina 2 mins after instillation of the semen and immediately assessed for sperm motility. Across the 6 animals tested in a blinded fashion, both 333 µg and 33 µg of LamH10 agglutinated >99% of PM sperm, while 3.3 µg agglutinated on average ≈90% of PM sperm (Figure 5B). These results confirm that LamH10 possesses markedly greater potencies than HCA IgG and also the decavalent FFIFF,^[^ ^21^ ^]^ underscoring the potentially very strong contraceptive potencies of LamH10 *in humans*.

### 2.7 LamH10 is Stable After Lyophilization and Formulation into Fast‐Dissolving Antibody Tablets (FDAT) for On‐Demand Non‐Hormonal Contraception

Next, we assessed the suitability of LamH10 for formulation into a tablet as a means of on‐demand contraceptive delivery. LamH10 was lyophilized in the presence of trehalose, then pressed into a 6‐mm round FDAT with a hydraulic press (Figure 5C). The FDAT rapidly dissolves upon exposure to moisture, taking 2 min to dissolve upon exposure to CVM (Figure 5D). LamH10 maintained agglutination potency after reconstitution either from lyophilized powder or FDAT, evaluated by comparing the agglutination potency against purified sperm to the original LamH10 solution (Figure 5E).

Finally, we used the same sheep pseudo‐PCT model to assess the potency of LamH10‐FDAT. Here, sheep received either PBS, a placebo FDAT, or a LamH10 FDAT (Figure 5F), underwent a brief simulated intercourse, followed by semen inoculation 5 min after the initial FDAT insertion, and assessing PM sperm fractions 2 min later. Studies were once again executed in a blinded fashion. Compared to PBS, the placebo FDAT slightly reduced PM sperm by ≈34%. In contrast, LamH10‐FDAT reduced PM sperm by 100% across all tested sheep (Figure 5G).

## 3 Discussion

mAb‐mediated potent non‐hormonal contraception has been a long‐sought goal for decades. Motivated by the discovery of immune infertility, the first attempts were based on contraceptive vaccines, which suffered from variable effectiveness and the risk of permanent infertility. As mAb bioprocessing began to mature, the efforts then transitioned to harnessing anti‐sperm IgG mAbs to enable transient contraception. Given the limited agglutination potencies of conventional IgGs, there is intense interest in engineering more potent mAbs. Our work here represents the latest and most potent iteration of engineered mAbs for non‐hormonal contraception, with LamH10 capable of achieving over 400‐fold greater agglutination potencies than the predicate HC4‐IgG, while maintaining exceptional stability at low pH and low concentrations. These properties make the LamH platform promising for the development of monospecific multivalent Abs where agglutination is a critical effector function. The controlled assembly of up to 3 unique protein chains in the trimer also allows for multivalent and multispecific applications, if single‐domain binders are used.

The improved performance of LamH*n* constructs over FFIFF and RGY at 37°C highlights essential features on multivalent ASAs, including stability, Fab presentation, and valency, as well as linker selection. For instance, LamH6 is similar to RGY in performance, despite halving the number of Fab domains per molecule. This is likely due to the covalent stabilization of the laminin trimerization domains, which minimizes concentration and pH‐dependent disassembly of the multivalent molecules. All LamH*n* constructs, as well as RGY, outperform FFIFF, which appears to confirm our hypothesis that the fusion of the Fab at the N‐terminus in FFIFF is detrimental to the binding of the H6‐C34 Fab. This is further supported by the slightly lower performance of LamH12 compared to LamH10 despite two additional Fab: in LamH12, the additional Fabs can only be added by fusing to the C‐terminus of the alpha domain, located to the C‐terminus of the Fc, and thus occupy the N‐terminus of the additional Fabs. Thus, LamH12 actually contains fewer available Fab per µg of protein compared to LamH10. Finally, the enhanced performance of LamH constructs using a hinge linker compared to a glycine‐serine linker underscores the importance of selecting suitable linkers in maximizing multivalent binding. We speculate this is due to an entropic penalty from the higher flexibility of glycine‐serine linkers; after a Fab unbinds, the more flexible GS linkers likely allow the Fab domain to more quickly diffuse away from the previously bound epitope, thus reducing the probability of rapid rebinding. This in turn decreases the net number of bonds that GS‐containing molecules can make with sperm at steady state, making it easier for sperm to escape agglutinates.

In addition to the orders of magnitude improvement in the sperm agglutination potency of LamH10, the exceptionally low dose (tens of micrograms) needed to achieve complete agglutination in sheep also likely reflects the superior biodistribution with direct vaginal delivery that makes the entire dose available at the site of action. Systemic delivery dilutes the dose into a large blood volume, which distributes most of the dose to non‐target tissues, and results in a substantial delay before ASA can be distributed into the FRT. Instead, the low total volume of secretions within the FRT (≈1mL or less^[^ ^56^ ^]^) implies that even relatively small total amounts of mAb can achieve a high local concentration. Finally, local delivery also likely improves safety, both because mAbs delivered to mucosal surfaces like the FRT is poorly absorbed into systemic circulation,^[^ ^57^, ^58^ ^]^ and also because vagina is a poorly immune inductive site with limited immune responses even when inoculating antigens in the presence of highly immunostimulatory adjuvants.^[^ ^59^ ^]^

In the past, high costs of mAb production and a focus on systemic delivery have limited the development of ASAs as a contraceptive strategy. Local delivery combined with declining costs due to advances in efficiency with CHO‐based manufacturing^[^ ^60^, ^61^, ^62^ ^]^ over the past decades means that ASAs may soon be a cost‐effective contraceptive alternative. We anticipate that multivalent formats, such as LamH10, that can reduce the total dose needed will help further drive down costs. Additionally, LamH10 provides the benefit of covalent assembly of the protein chains, improving stability and homogeneity compared to formats such as RGY.

Sperm must swim through mucus to ascend into the upper reproductive tract and eventually reach the egg. Of ≈200 million total sperm in the ejaculate, only ≈1% of the spermatozoa enter the cervix, and only a few dozen might reach the egg.^[^ ^63^ ^]^ While low sperm count alone is not predictive of low fertility,^[^ ^64^, ^65^, ^66^ ^]^ both poor sperm motility in cervical mucus and reduced total sperm count contribute to a high likelihood of infertility.^[^ ^67^ ^]^ Human semen typically contains 45–65 million sperm mL^−1^, while a concentration below 5 million sperm mL^−1^ is considered severely oligospermic and is associated with low fertility.^[^ ^68^ ^]^ Similarly, azoospermia and oligospermia induced by testosterone, which does not affect sperm morphology^[^ ^69^ ^]^ or in vitro fertilizing capacity,^[^ ^70^ ^]^ exhibit very low fertility rates at 0.8 conceptions per 100 women‐years at concentrations below 1 million sperm mL^−1^.^[^ ^66^, ^71^, ^72^ ^]^ Together, this suggests that a substantial reduction of progressive sperm motility, even if incomplete, has the potential to provide effective contraception.

There are several limitations of the current study. Importantly, we were unable to directly demonstrate pregnancy prevention as the target antigen is present only on human and chimpanzee sperm,^[^ ^49^ ^]^ and chimpanzee studies are not possible in the US. Instead, we used an in vivo sheep model closely mimicking the human PCT test as a proof‐of‐concept. Efficacy in humans will need to be rigorously evaluated in clinical studies. A recent Phase 1 clinical trial of the parent IgG has shown promising initial safety and reduction of progressively motile sperm when delivering a relatively high dose of 20mg in a vaginal film.^[^ ^29^ ^]^ Here, we observed very promising efficacy when delivering a dose of 1mg via tablet in sheep, and delivery in solution suggests that LamH10 remains effective even at doses as low as 33µg. This may allow for sustained delivery of Ab in other formats such as a vaginal ring in future studies.

## 4 Experimental Section

### Ethics Statement

Human semen and human cervicovaginal mucus (CVM) samples used for in vitro studies at UNC were collected under a protocol approved by the Institutional Review Board (IRB) of the University of North Carolina (UNC) at Chapel Hill (IRB #10‐1817). Human semen and human CVM used in site assessments at Mucommune LLC were collected under a protocol approved by an independent IRB through IRB Solutions (IRB# 0112). Informed written consent was obtained from all male and female subjects prior to the collection of semen and CVM samples. Human semen samples used in the sheep surrogate PCT study were collected under a protocol (IRB# 18–0254) approved by the IRB of the University of Texas Medical Branch (UTMB). All male volunteers provided informed written consent prior to sample collection. Sheep studies using six female Merino crossbred sheep followed protocols approved by the UTMB Institutional Animal Care and Use Committee (IACUC# 06–08038D).

### Semen Collection and Purification of Motile Sperm

Health male subjects between 18–45 years old were asked to collect semen by masturbation into sterile 50 mL sample cups. Subjects were asked to refrain from sexual activity at least 48 h prior to sample collection. Samples were incubated at least 15min after collection at room temperature (RT), or until liquefaction. Samples that did not liquefy were discarded. Density gradient centrifugation was used to separate the motile sperm fraction from liquefied ejaculate. A two‐layer gradient was prepared by layering a 1.5 mL of Isolate 50% density medium over a 1.5mL Isolate 90% density medium (Irvine Scientific, #99264). Up to 3mL of ejaculate was carefully layered on top of the gradient, then centrifuged at RT at 300xg for 20min. After centrifugation, the upper layers containing seminal plasma and dead cells were removed, taking care not to disturb the pellet of motile sperm at the bottom. The sperm pellet was then washed two times with sperm multipurpose handling medium (MHM) (Irvine Scientific, #90166). The washed pellet was then resuspended in MHM and assessed for count and mobility using CASA. Samples were discarded if they did not meet the lower reference limit for total motility (40%) and sperm count (15×10^6^ sperm mL^−1^) indicated by World Health Organization guidelines.^[^ ^73^ ^]^ The baseline CASA counts and motility characteristics of all samples used in the study post‐purification are listed in Table S1 (Supporting Information).

### CASA Sperm Count and Motility Analysis

Hamilton Thorne CASA version 12.3 was used in all experiments unless stated otherwise. For each sample, 2 chambers of a 20µm depth sperm counting slide (Leja 4‐Chamber slides) were filled with 3 µL of the sample. In each chamber, 10 randomly selected microscopic fields near the slide center were imaged and averaged for count and motility. The assessments of the 2 chambers were then averaged. The complete set of parameters used by the Hamilton Thorne Ceros 12.3 software is listed in Table S2 (Supporting Information). Sperm were defined as progressively motile if they had a VAP of at least 25µm s^−1^ and 80% STR.^[^ ^74^ ^]^

### CVM Collection and Processing

CVM was collected as previously described.^[^ ^75^ ^]^ Briefly, undiluted CVM was self‐sampled from women of reproductive age, ranging from 20 to 33 years old, using a disposable menstrual cup (Instead Softcup). Participants inserted the device into the vagina for at least 60s, twisted while removing, and placed the ring into a 50‐mL conical tube. Samples were centrifuged at 500xg for 5min to collect the secretions off the ring. The samples averaged 0.3g per sample. αunprotected intercourse before providing the sample. All samples were collected a minimum of 72h after the reported end of the last menstrual cycle.

### Construction of LamH Construct Plasmids

The variable light (V_L_) and variable heavy (V_H_) nucleotide sequences of the published sequence of H6‐3C4^[^ ^24^, ^76^ ^]^were used in all constructs. Each protein chain was cloned into the pαH mammalian expression vector as the backbone. The trimerization domains were obtained from the E8 domain of laminin‐511.^[^ ^30^ ^]^ Each construct required a light chain (LC), two mid‐length chains (MC) containing β and γ trimerization domains, and a heavy chain (HC) containing the α trimerization domain and Fc region of the antibody. The LC and two MCs were identical across constructs. The linker used was the upper hinge region of IgG_1_ antibody, amino acid sequence EPKSCDKTHT. The LC was constructed in the format V_L_‐C_λ_ domains. The β chain was expressed in the format V_H_‐CH_1_‐Linker‐β trimer domain. The γ chain was expressed in the format V_H_‐CH_1_‐Linker‐γ trimer domain. The HCs were expressed in the formats V_H_‐CH_1_‐Linker‐α trimer domain‐CH_2_‐CH_3_, V_H_‐CH_1_‐Linker‐α trimer domain‐CH_2_‐CH_3_‐Linker‐ α trimer domain, and V_H_‐CH_1_‐Linker‐α trimer domain‐CH_2_‐CH_3_‐Linker‐ α trimer domain‐Linker‐ V_H_‐CH_1_ for LamH6, LamH10, and LamH12, respectively.

### Expression and Purification of Ab

Plasmids expressing LamH constructs or the parent IgG were co‐transfected into Expi293F cells using ExpiFectamine293 transfection reagents (Gibco, Gaithersburg, MD). For IgG and RGY, HC and LC plasmids were co‐transfected at a 1:1 ratio following the manufacturer's protocol. For LamH6, LamH10, and LamH12, α HC, β MC, γ MC, and LC were co‐transfected in a 1:2:2:5 ratio at 2 µg total DNA per 1 mL culture. Transfected cells were grown at 37° in a 5% CO_2_ incubator while shaken at 125 rpm for 3–5days, or until viability dropped below 60%. The culture supernatant was then harvested by centrifugation for 10min at 1000xg and passed through 0.22µm vacuum filters. Abs were then purified using protein A chromatography. 120mL of culture supernatant was mixed with 250µL of Pierce protein A plus resin (Thermofisher Scientific, #53142), then split into 50mL conical tubes and incubated at 4°C overnight with end‐over‐end mixing. The supernatant was then loaded into 20mL Econo‐Pac® Chromatography Columns (Bio‐rad, #732101) until all resin was collected. The resin was then washed three times with 5mL 1x phosphate‐buffered saline (PBS). Protein was then eluted from the column using 900µL Pierce IgG Elution Buffer (Thermofisher Scientific, #21004), which was immediately neutralized with 100µL 1M Tris‐HCl, pH 8.0 (Thermofisher Scientific, #15568025). Purified Ab was quantified by nanodrop using absorbance at 280nm, using the molecular weight and extinction coefficient of each protein. The molecular weights of LamH6, LamH10, and LamH12 were calculated as 403kDa, 655kDa, and 753kDa, respectively. The extinction coefficients were calculated as 606220 cm^−1^ M^−1^, 972240 cm^−1^ M^−1^, and 1142440 cm^−1^ M^−1^ respectively.

### Biophysical Characterization of Abs

The relative size of unreduced purified constructs and the molecular weight of their component protein chains were determined using sodium dodecyl sulfate‐polyacrylamide gel electrophoresis (SDS‐PAGE) in reducing and non‐reducing conditions. For each sample, 1–2 µg of protein was added to 3.75 µL NuPAGE^TM^ lithium dodecyl sulfate (LDS) sample buffer (Thermo Scientific, #NP0007). In reduced‐condition aliquots, 1 µL of 0.5 M tris (2‐carboxyethyl) phosphine (TCEP) (Thermo Scientific, #T2556) was added as the reducing agent. Aliquots were then filled with nuclease‐free water to a final volume of 15 µL. Samples were then denatured by holding in a thermocycler at 70°C for 10min. Samples were loaded into a NuPAGE^TM^ 3–12% Bis‐Tris gel (Thermo Scientific, #NP0322), and Novex^TM^ Sharp Pre‐stained Protein Standard(Thermo Scientific, #LC5800) was used as the standard. The gel was run for 1 hr at 200V, then stained for 1 hr with Imperial Protein Stain (Thermo Scientific, #24615) before being destained overnight in double‐distilled water.

Size exclusion chromatography with multi‐angle light scattering (SEC‐MALS) was employed to evaluate the molecular weight of the unreduced purified Abs and the overall homogeneity of the Ab solutions. A Bio‐Rad FPLC system with a GE Superdex 200 10/300 column was connected to a Wyatt miniDAWN MALS instrument. Experiments were run at 4°C with a flow rate of 0.25 mL min^−1^. The column was equilibrated with sterile‐filtered 1x PBS pH 7.4, then 100µL of each sample at a concentration of 1–2mg mL^−1^ was injected onto the column. MALS data were analyzed using Wyatt ASTRA 8 software.

The T_m_ and T_agg_ of the purified Abs were determined via nano differential scanning fluorimetry (nanoDSF) using a Nanotemper Prometheus NT.48 system.^[^ ^77^ ^]^ Samples were diluted to 0.5mg mL^−1^ in 1X PBS, pH7.4, then loaded into Prometheus NT.48 capillaries. Each sample was evaluated in triplicate. Thermal denaturation experiments were executed over the range of 20°C to 95°C increasing at 1°C min^−1^, while measuring intrinsic tryptophan fluorescence at 330nm and 350nm. The T_m_ was calculated automatically by the manufacturer's software from the first derivative of the ratiometric measurement of these fluorescent signals as temperature increases. Similarly, the T_agg_ is calculated by the manufacturer's software based on measurements of back‐reflected light from a light beam passed through the sample at each temperature. As the Ab aggregates, more light is scattered, and the back‐reflection of light is reduced.

### Whole Sperm Enzyme‐Linked Immunosorbent Assay (ELISA)

The binding affinity of each construct was evaluated by ELISA against whole sperm. Motile sperm were purified from whole semen as described above, then coated onto half‐area high‐binding polystyrene plates (CLS3690, Corning) at 2×10^5^ sperm per well in 50 µL of NaHCO_3_ buffer, pH 9.6. Plates were incubated overnight at 4°C, then centrifuged at 300xg for 20min. The supernatant was decanted, then the plate was air‐dried for 1 hr at 45°C. Plates were washed 3x with PBS, then blocked with 150µL per well of 5% milk at RT for 1 hr. After an additional 3x wash with PBS, Abs were diluted in 1% milk at a starting concentration of 100nM, then serially diluted at fivefold intervals and plated in triplicate at 50 µL per well. Plates were incubated for 1 hr at either RT or 37°C. Plates were washed again 3x with PBS, which was either at RT or had been pre‐warmed to 37°C according to the incubation temperature of the prior step. Afterwards, plates were incubated for 1 hr at RT with 50µL per well of HRP‐conjugated goat anti‐human IgG Fc (Abcam, # ab98624) diluted 1:10,000 in 1% milk. The washing procedure was repeated, and then the plate was developed by incubating 50 µL per well of 1‐Step Ultra TMB ELISA Substrate (Thermofisher Scientific, #34028) in the dark at RT for 15 min. The reaction was then quenched using 50 µL per well of 1N HCl. The absorbance at 450 nm (signal) and 570 nm (background) was measured with a SpectraMax M2 Microplate Reader (Molecular Devices).

### Agglutination Kinetics Assay

Purified sperm resuspended at an initial concentration of 40 × 10^6^ sperm mL^−1^ in sperm multi‐handling media. A sperm counting assessment chamber slide (Leja, 4‐chamber, 20µm depth) was pre‐warmed on a MiniTherm® stage warmer set to 37°C. Sperm stock and antibody dilutions were held at 37°C in a water bath. Then, 6 µL of purified sperm was mixed 1:1 (working concentration 20 × 10^6^ sperm mL^−1^) with 6 µL of Ab dilution in 0.2 mL PCR tubes, moving rapidly to minimize cooling time. A timer was started immediately upon initiating mixing, then 3 µL of sperm Ab‐mixture was transferred to the chamber slide. The centerfield of the slide was then imaged and analyzed by CASA at 30, 60, and 90s. The reduction in PM sperm at each point was computed by normalizing to the PM sperm count in a control slide mixed with sperm handling medium alone. Data was obtained from n = 6 samples, all from unique donors, and each sample was assessed in duplicate, and the average of the two chambers was taken. The reduction in the number of tracked PM sperm was used as a proxy measurement for overall agglutination, as sperm captured by agglutination were unable to achieve progressive motility. As agglutination makes the total sperm count under CASA unreliable due to the inability to distinguish all sperm in an agglutinated cluster, reduction in motility was calculated only through comparing the number of progressive tracks between conditions, as unagglutinated sperm could still be reliably tracked. The assessment was also repeated for each donor using unaltered whole semen in place of purified sperm using the selected candidate Ab LamH10. Assessments were started at 10 µg mL^−1^ when working with purified sperm or 40 µg mL^−1^ when using whole semen, and fourfold dilutions were assessed until complete failure of the LamH10 was observed.

### Sperm Escape Assay

Whole semen was placed into a pcr tube, and an equal volume of media containing the ASA was layered gently on top. The sample was then held at a 45° angle at 37°C for 5 min. A sample was taken from the topmost section of the media and evaluated via CASA for the quantity of PM sperm.

For each sample, 40 µL aliquots of whole semen were transferred to individual 0.2 mL PCR tubes. Abs were diluted in sperm handling medium, and subsequently, 40 µL of either untreated medium or an Ab dilution was layered gently on top of the whole semen. The tubes were then held in a thermocycler propped at a 45° angle at 37°C for 5 min. Following this incubation, 6 µL was extracted from the top layer and transferred to a chamber slide held in a stage warmer. The number of PM sperm was summed over 10 fields of view in each condition, and the percentage decrease was calculated by normalizing to the untreated control. The data represent 6 independent experiments from 6 unique sample donors. Each experimental condition was assessed in duplicate on each sample. The Abs were assessed starting at 40 µg mL^−1^ and working down with fourfold serial dilutions.

### Fluorescent Sperm Labeling

SYBR14 dye, a membrane‐permanent nucleic acid stain, was used to stain live sperm. Motile sperm were isolated from whole semen as described above. The purified sperm was incubated with SYBR14 from a Live/Dead Sperm Viability kit (Thermo Scientific, #L7011) at a final concentration of 200nM for 10min in a water bath at 37°C. The labeled sperm was then washed 3 times with sperm washing medium after centrifuging at 300xg for 10 min to remove unbound fluorophores. The pellet was then resuspended in 1mL of sperm handling medium and assessed for count and motility via CASA.

### Muco‐Trapping Assessment

The muco‐trapping potency of each antibody was assessed as previously described[ref]. Briefly, multiple particle tracking of fluorescently labeled sperm was performed at sub‐agglutinating concentrations of sperm. Fresh CVM was titrated to pH 6.8–7.1 using small volumes of 3N NaOH, then diluted threefold using sperm washing medium to mimic the dilution of CVM alkaline seminal fluid. Next, 100 µL of the diluted and pH‐adjusted CVM was aliquoted into chambers of a CultreWell^TM^ chamber slide (Invitrogen, Thermo Scientific #C37000). Next, Ab was added to a final concentration of 10 µg mL^−1^ and mixed well within the chamber using a wiretrol. Finally, sperm was added to a final concentration of 2×10^4^ sperm mL^−1^. Translational motions of the fluorescently labeled sperm were recorded using an electron‐multiplying charge‐coupled‐device camera (Evolve 512; Photometrics, Tucson, AZ) mounted on an inverted epifluorescence microscope (AxioObserver D1; Zeiss, Thornwood, NY) equipped with an Alpha Plan‐Apo 20/0.4 objective, environmental (temperature and CO2) control chamber, and light‐emitting diode (LED) light source (Lumencor Light Engine DAPI/GFP/543/623/690). Videos (512 × 512 pixels, 16‐bit image depth) covering at least 10 fields of view and 50 total tracked sperm were captured for each condition using MetaMorph imaging software (Molecular Devices, Sunnyvale, CA) at a temporal resolution of 66.7 ms and spatial resolution of 50 nm (nominal pixel resolution, 0.78 µm pixel^−1^) for 10s. Assessments of the parent IgG were done at room temperature to allow sufficient binding. Convolutional neural network‐based tracking software was used to determine x, y coordinates of each sperm in each video frame.^[^ ^78^ ^]^ These coordinates were used to calculate quantitative metrics commonly used to describe sperm motion, such as velocity of the average path (VAP), curvilinear velocity (VCL), straight‐line velocity (VSL), and straightness (STR). As sperm did not swim as quickly in CVM as in buffer or semen, in which these characteristics were commonly assessed, VSL and STR were relied upon to classify progressive sperm in place of VAP and VCL. Based on visual inspection of classification success in a subset of videos, sperm were marked progressive if they exhibited at least 3µm s^−1^ VSL and 0.8 STR. Data represent 7 independent experiments on unique semen‐CVM pairs, composed of 5 unique semen specimens and 4 unique CVM specimens.

### Formulation of FDAT

LamH10 was lyophilized in histidine buffer (50mM, pH 6.5) with 46% by weight trehalose. For each tablet, 1.92mg (1mg Ab, 0.92mg trehalose content) of the lyophilized powder was then mixed with 5mg sodium bicarbonate, 5mg citric acid, 19.04 mg erythritol, and 19.04mg microcrystalline cellulose for a total of 50mg. This mixture was ground into a fine powder with a mortar and pestle, then loaded into a custom die and punch. A customized hydraulic press (Carver) set at 0.03t was used to press the powder together into 6‐mm diameter tablets. LamH10 was evaluated for activity post‐ lyophilization and manufacture into tablets by reconstitution into PBS or vaginal fluid simulant then comparing agglutination via an agglutination kinetics assay as described above. Dissolution of the final tablet was assessed by placing a tablet into either 1mL of vaginal fluid simulant or 1mL pooled CVM, and imaging at 15s intervals.

### In Vivo Surrogate Efficacy Studies

On each test day, all sheep were dosed with the same semen mixture pooled from 3–5 donors, then received a randomized Ab treatment. Each Ab condition was assessed at least 3 times in the same group of sheep. Experiments were performed in 6 individual Merino crossbred sheep over 3 weeks, with 7 days between each treatment round. In the first two weeks, Ab was delivered in solution. Sheep received 1mL of solution containing 333 µg, 33 µg, or 3.3 µg of LamH10 or PBS alone, which was instilled into the vagina followed by mixing using a vaginal dilator for 15 strokes. Then, 1mL of pooled whole semen was pipetted into the sheep's vagina, followed by simulated intercourse with a vaginal dilator for 5 strokes. At 2 min after semen was introduced, fluids were collected and from the sheep vagina and assessed for progressive sperm count in a hemocytometer (Bright‐Line Hemacytometer) under a light microscope (Olympus IX71) using a 20× objective with Thorlabs camera. Last week, sheep received either a LamH10 tablet, a placebo tablet, or a PBS solution. The tablets were allowed to dissolve for 5 min, then the experiment proceeded following the same procedure as above. Treatments and quantifications were performed blinded.

### Statistical Analysis

All analyses were performed with GraphPad Prism software (v. 10.2.3). For multiple group comparisons involving two factors, P‐values were calculated using two‐way ANOVA with post‐hoc Holm‐Sidak test (Figures 1B and 3A,C). For multiple group comparisons involving one factor, p‐values were calculated using one‐way ANOVA with post‐hoc Tukey's test (Figures 2D and 5B). For the muco‐trapping analysis, repeated‐measures ANOVA was used with post‐hoc Holm‐Sidak test (Figure 4B–D). In all analyses, α = 0.05 was the threshold for statistical significance. The values for N, P, and the specific statistical test performed for each experiment were included in the appropriate figure legends. All data were presented as the mean ± SD.

## Conflict of Interest

S.K.L. is the founder of MUCOMMUNE, LLC, and currently serves as its interim CEO. S.K.L. is also the founder of Inhalon Biopharma, Inc., advancing the ‘muco‐trapping’ mAb platform for respiratory health, and currently serves as its CSO, Board of Directors, and Scientific Advisory Board. S.K.L. has equity interests in both Mucommune and Inhalon Biopharma; S.K.L.’s relationships with Mucommune and Inhalon are subject to certain restrictions under University policy. The terms of these arrangements are managed by UNC‐CH in accordance with its conflict‐of‐interest policies. A.S. and S.K.L. are inventors on a patent licensed by Mucommune and Inhalon Biopharma. K.K. is an employee of Mucommune, LLC and is an inventor on patent filed on vaginal tablet formulations.

## Supporting information



Supporting Information

## Data Availability

The data that support the findings of this study are available from the corresponding author upon reasonable request.
