# Correction to “Engineering Decavalent, Sperm‐Binding Laminin‐IgG Hybrid Antibodies for Potent Non‐Hormonal Contraception”

**DOI:** 10.1002/advs.202514968

**Published:** 2025-08-18

A. Schaefer, K. Kushiro, B. Shrestha, Y. Zhu, A. Panjwani, L. Dawson, H. Flowers, K. L. Vincent, S. K. Lai, Engineering Decavalent, Sperm‐Binding Laminin‐IgG Hybrid Antibodies for Potent Non‐Hormonal Contraception. *Adv. Sci*. 2025, e06272.

https://doi.org/10.1002/advs.202506272

The legends of Figure 3A,C were omitted. The figure should be as follows:


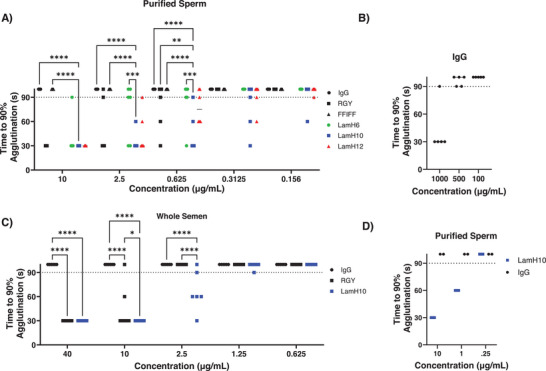


We apologize for this error.

