# Supplementary material for: Engineering Decavalent, Sperm‐Binding Laminin‐IgG Hybrid Antibodies for Potent Non‐Hormonal Contraception
Source: Adv Sci (Weinh). 2025 Jul 23;12(38):e06272. doi: 10.1002/advs.202506272 (PMC12520502; doi:10.1002/advs.202506272)
Supplement: Supplementary file 1 — Supporting Information [file ADVS-12-e06272-s001.docx]

**Supplementary Methods**

*Generation of trimerized constructs for screening*

The variable light (V_L_) and variable heavy (V_H_) nucleotide sequences of the published sequence of H6-3C4[^1^](https://sciwheel.com/work/citation?ids=14014227&pre=&suf=&sa=0&dbf=0) were used in all constructs. Each protein chain was cloned into the pαH mammalian expression vector as the backbone. The initially screened constructs were analogous to LamH6, where it was intended to display 6 Fabs, 3 trimerized on each arm of the IgG. The linker used between all globular domains was 6xGGGGS. Two homotrimer domains from collagen XV and XVIII were screened[^2^](https://sciwheel.com/work/citation?ids=9178191&pre=&suf=&sa=0&dbf=0). Each construct required a light chain (LC), a mid-length chain (MC) containing the trimerization domain, and a HC containing a trimerization domain and the Fc region of the Ab. The LC was constructed in the format V_L_-C_λ_ domains. The MC was expressed in the format V_H_-CH_1_-Linker- trimer domain. The HCs were expressed in the formats V_H_-CH_1_-Linker- trimer domain-CH_2_-CH_3_. We additionally screened a heterotrimer domain from the NC1 portion of collagen IV[^3^](https://sciwheel.com/work/citation?ids=11548836&pre=&suf=&sa=0&dbf=0). This trimer is composed of two α1 chains and one α2 chain. This construct required plasmids for an LC, an MC containing the α1 trimerization domain, and an HC containing the α2 trimerization domain and Fc region of the Ab. The LC was constructed in the format V_L_-C_λ_ domains. The MC was expressed in the format V_H_-CH_1_-Linker- α1 trimer domain. The HC was expressed in the format V_H_-CH_1_-Linker- α2 trimer domain-CH_2_-CH_3_. The last trimer domain screened was a computational designed trimer known as DHT03[^4^](https://sciwheel.com/work/citation?ids=16604003&pre=&suf=&sa=0&dbf=0), which requires 3 unique A, B, and C trimer domains. This construct required plasmids for an LC, an MC containing the A trimerization domain, an MC containing the B trimerization domain and an HC containing the C trimerization domain and Fc region of the Ab. The LC was constructed in the format V_L_-C_λ_ domains. The MCs were expressed in the formats V_H_-CH_1_-Linker- A trimer domain and V_H_-CH_1_-Linker- B trimer domain. The HC was expressed in the format V_H_-CH_1_-Linker- C trimer domain-CH_2_-CH_3._

*Stability in CVM assay*

Either LamH10 or the parent IgG was diluted into 100 µl whole, native CVM to a final concentration of 160 µg/mL. The native pH was measured via micro pH meter and recorded; native pH ranged from 3.8-4.2. Aliquots were incubated for 18h overnight at either 37°C or 4°C. The following day, the CVM-Ab mixtures were diluted with 100 µl PBS and mixed thoroughly by vortexing. The mucins were then spun out by centrifugation at 15,000g for 10 min, and the supernatant harvested. The supernatant was treated as having an initial concentration of 80 µg/mL of Ab. These were then assessed for agglutination activity by the agglutination kinetics assay against whole semen.

*Long-term temperature stability assay*

LamH10 was prepared at 5 mg/mL concentration in 20mM Histidine, 100mM Arginine, pH 6.5. The Ab was split into 10 0.2 mL aliquots. Half of the aliquots were stored in 4°C as controls, and the other half was stored in a 37°C incubator for 28 days. At each time point: Day 0, Day 7, Day 14, Day 21, and Day 28, one aliquot was taken out from both 4°C and 37°C for the characterization using SEC-MALS, SDS-PAGE, and Agglutination Kinetics assay against purified sperm.

**Supplementary Table S1. Baseline Motility Characteristics of Purified Sperm Samples**

| **Sample ID** | **Sperm Concentration (10E6/mL)** | **%Progressively Motile** |
| --- | --- | --- |
| M55-0802 | 17.6 | 41.8 |
| M38-0802 | 22 | 42.8 |
| M55-0805 | 34.8 | 46.3 |
| M46-0805 | 21.4 | 44.4 |
| M41-0402 | 22.5 | 44.2 |
| M55-0422 | 19.2 | 47.5 |
| M41-0422 | 22.5 | 50.8 |
| M54-0318 | 31 | 54.2 |
| M45-0418 | 15.7 | 46 |
| M55-0320 | 22.6 | 48.4 |
| M45-0404 | 22.5 | 66 |
| M53-0318 | 42.6 | 50 |
| M50-0311 | 17.8 | 52.3 |
| M43-0313 | 27.2 | 44 |
| M54-0412 | 19.5 | 47 |

**Supplementary Table S2. Motility Parameters of Hamilton-Thorne Ceros 12.3**

| Parameter | Value | Parameter | Value |
| --- | --- | --- | --- |
| Frames Per Second | 60 | Path Velocity (VAP) | 25 µm/s |
| Number of Frames | 60 | Straightness (STR) | 80% |
| Minimum Cell Size | 3 pixels | VAP Cutoff | 10 µm/s |
| Default Cell Size | 6 pixels | VSL Cutoff | 0 µm/s |
| Minimum Contrast | 80 | Standard Objective | 10X |
| Default Cell Intensity | 20 | Magnification | 1.87 |
| Chamber Depth | 20 µm | Slow Cells | Motile |

**Supplementary Figure S1. Screening of trimerized Ab constructs via SEC-MALS.** **A)** Ab trimerized using the homotrimer domain from Collagen XV. **B)** Ab trimerized using the homotrimer domain from Collagen XVIII. Both constructs using a homotrimer domain were able to assemble in two distinct populations, with only the smaller population corresponding to the correct size of the desired 6-Fab display format. **C)** Construct constructed with a heterotrimer domain from collagen IV. **D)** Construct expressed with a computationally designed obligate heterotrimer DHT03. Both heterotrimers expressed a primary population too small to be the assembled 6-Fab display format.

**Supplementary Figure S2. SEC-MALS UV and Scattering Data of parent IgG, LamH6, LamH10, and LamH12.**

**Supplementary Figure S3. Room Temperature ELISA Assessment of LamH10**

**Supplementary Figure S4. Kinetics of sperm agglutination in purified sperm.** The kinetics of sperm agglutination across mAb concentrations, as determined by the relative reduction in PM sperm fractions at 30 second intervals following addition of mAbs to purified sperm, compared to sperm-handling media negative control. Data points and error bars represent mean and standard deviation from n=6 independent experiments with semen samples from unique donors. Measurements in each sample were performed in duplicate and averaged.

**Supplementary Figure S5.** Minimum Dose Required to achieve at least 90% reduction in progressively motile sperm within 90s in either A) purified sperm (n=6 samples) or B) whole semen (n=6 samples). Dotted lines indicate the highest time or concentration tested, and points above represent samples which did not achieve 90% agglutination under any tested condition.

**No Ab +0.625 µg/mL LamH10**

**
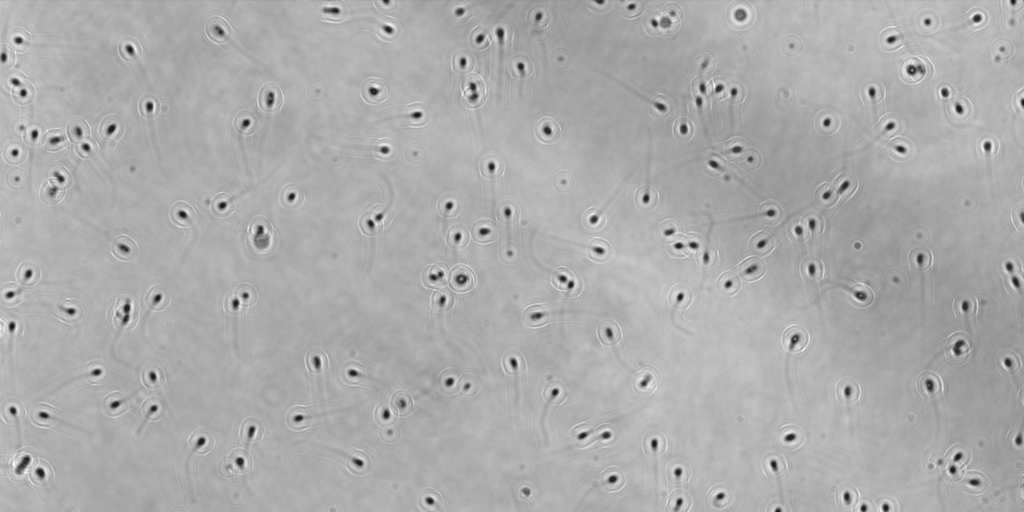
**
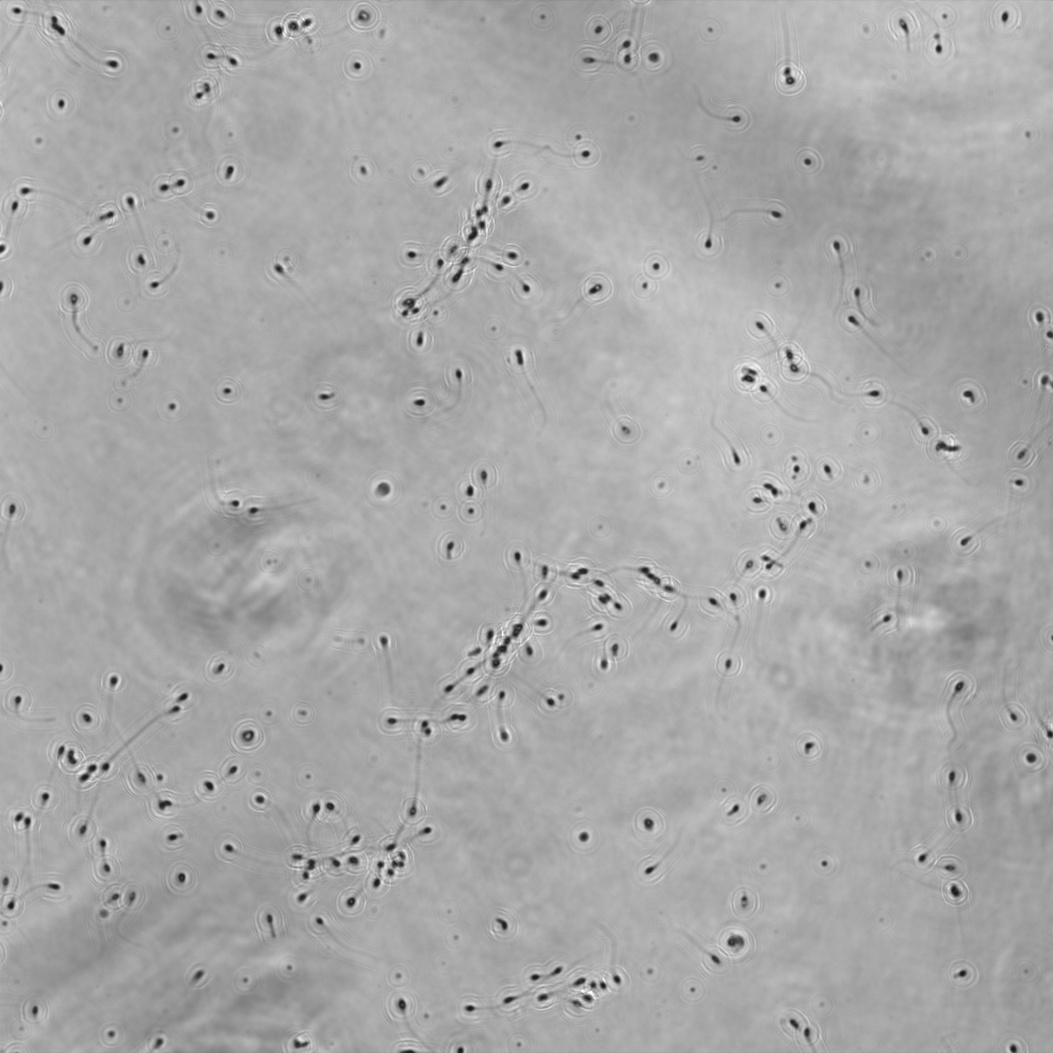


**Supplementary Figure S6. Brightfield images of purified sperm either with or without 1ug/mL of LamH10.**

**Supplementary Figure S7. The kinetics of sperm agglutination across mAb concentrations in whole semen**. Measured by the relative reduction in PM sperm fractions at 30 second intervals following addition of mAbs to whole semen, compared to sperm-handling media negative control. Data points and error bars represent mean and standard deviation from n=6 independent experiments with semen samples from unique donors. Measurements in each sample were performed in duplicate and averaged.

**Supplementary Figure S8. Stability Assessments of LamH10 at 37°C and pH 4. A)** Agglutination potency in whole semen of IgG and LamH10 after 18h incubation in whole human CVM at either 37°C or 4°C. Data obtained from n=3 unique CVM samples, each of which were paired with at least 2 unique semen samples. **B)** Agglutination potency of LamH10 during long-term exposure to 37°C conditions, at 7-day intervals up to 28 days. **C)** SDS-Page of LamH10 at 7-day intervals of incubation at 37°C. **D)** Monomer content of LamH10 after incubation at 37°C as measured by SEC-MAls, at 7-day intervals to 28days. **E)** Assessment of stability of LamH10 at 5mg/mL and 0.5 mg/mL at pH7, and 5mg/mL at pH4, as measured by comparison of UV-signal during SEC. P-values were obtained by two-way ANOVA with post-hoc Dunnett’s test. *P<0.05, ****P<0.001. Lines in panel A represent the median, Lines and error bars in panel B indicate arithmetic mean values and standard deviation.

**Supplementary Figure S9. Reduction of sperm escape in whole semen upon exposure to media containing various mAbs**. **(A)** Fraction of PM sperm compared to no mAb control at decreasing concentrations of HC4-IgG, HC4-RGY and LamH10. Data represents n=6 independent experiments from semen samples from unique donors. **(B)** Averaged reduction in escaped PM sperm across mAb concentrations. **(C)** Comparison of reduction in sperm escape at RT *vs.* 37°C for HC4-IgG, HC4-RGY, and LamH10. Data points represent mean and standard deviation from n=5 independent experiments of semen samples from unique donors. Experiments in each sample were performed in duplicate and averaged. P-values were obtained by two-way ANOVA with post-hoc Tukey’s multiple comparisons test. **P<0.01, ***P<0.001, ****P < 0.0001. Lines represent the median value in panel A; lines and error bars indicate arithmetic mean values and standard deviation in panels B and C.

**Reference**

[1.    Isojima, S. *et al.* Establishment and characterization of a human hybridoma secreting monoclonal antibody with high titers of sperm immobilizing and agglutinating activities against human seminal plasma. *J. Reprod. Immunol.* **10**, 67–78 (1987).](https://sciwheel.com/work/bibliography/14014227)

[2.    Alvarez-Cienfuegos, A. *et al.* Intramolecular trimerization, a novel strategy for making multispecific antibodies with controlled orientation of the antigen binding domains. *Sci. Rep.* **6**, 28643 (2016).](https://sciwheel.com/work/bibliography/9178191)

[3.    Burbelo, P. D., Martin, G. R. & Yamada, Y. Alpha 1(IV) and alpha 2(IV) collagen genes are regulated by a bidirectional promoter and a shared enhancer. *Proc Natl Acad Sci USA* **85**, 9679–9682 (1988).](https://sciwheel.com/work/bibliography/11548836)

[4.    Bermeo, S. *et al.* De novo design of obligate ABC-type heterotrimeric proteins. *Nat. Struct. Mol. Biol.* **29**, 1266–1276 (2022).](https://sciwheel.com/work/bibliography/16604003)
